# A Cross-Sectional Study of Water, Sanitation, and Hygiene-Related Risk Factors for Soil-Transmitted Helminth Infection in Urban School- and Preschool-Aged Children in Kibera, Nairobi

**DOI:** 10.1371/journal.pone.0150744

**Published:** 2016-03-07

**Authors:** Caitlin M. Worrell, Ryan E. Wiegand, Stephanie M. Davis, Kennedy O. Odero, Anna Blackstock, Victoria M. Cuéllar, Sammy M. Njenga, Joel M. Montgomery, Sharon L. Roy, LeAnne M. Fox

**Affiliations:** 1 Division of Parasitic Diseases and Malaria, Centers for Disease Control and Prevention, Atlanta, Georgia, United States of America; 2 Centre for Global Health Research, Kenya Medical Research Institute, Nairobi, Kenya; 3 Waterborne Disease Prevention Branch, Centers for Disease Control and Prevention, Atlanta, Georgia, United States of America; 4 Eastern and Southern Africa Centre of International Parasite Control, Kenya Medical Research Institute, Nairobi, Kenya; 5 Division of Global Health Protection, Centers for Disease Control and Prevention, Nairobi, Kenya; Brighton and Sussex Medical School, UNITED KINGDOM

## Abstract

Soil-transmitted helminth (STH) infections affect persons living in areas with poor water, sanitation, and hygiene (WASH). Preschool-aged children (PSAC) and school-aged children (SAC) are disproportionately affected by STH infections. We aimed to identify WASH factors associated with STH infection among PSAC and SAC in Kibera, Kenya. In 2012, households containing a PSAC or SAC were randomly selected from those enrolled in the International Emerging Infections Program, a population-based surveillance system. We administered a household questionnaire, conducted environmental assessments for WASH, and tested three stools from each child for STH eggs using the Kato-Katz method. WASH factors were evaluated for associations with STH infection using univariable and multivariable Poisson regression. Any-STH prevalence was 40.8% among 201 PSAC and 40.0% among 475 SAC enrolled. Using the Joint Monitoring Programme water and sanitation classifications, 1.5% of households reported piped water on premises versus 98.5% another improved water source; 1.3% reported improved sanitation facilities, while 81.7% used shared sanitation facilities, 13.9% had unimproved facilities, and 3.1% reported no facilities (open defecation). On univariable analysis, STH infection was significantly associated with a household toilet located off-premises (prevalence ratio (PR) = 1.33; p = 0.047), while always treating water (PR = 0.81; p = 0.04), covering drinking water containers (PR = 0.75; p = 0.02), using clean towels during hand drying (PR = 0.58; p<0.01), having finished household floor material (PR = 0.76; p<0.01), having electricity (PR = 0.70; p<0.01), and increasing household elevation in 10-meter increments (PR = 0.89; p<0.01) were protective against STH infection. On multivariable analysis, usually versus always treating water was associated with increased STH prevalence (adjusted prevalence ratio (aPR) = 1.52; p<0.01), while having finished household floor material (aPR = 0.76; p = 0.03), reported child deworming in the last year (aPR = 0.76; p<0.01), and 10-meter household elevation increases (aPR = 0.89; p<0.01) were protective against infection. The intersection between WASH and STH infection is complex; site-specific WASH interventions should be considered to sustain the gains made by deworming activities.

## Introduction

The World Health Organization (WHO) estimates that more than 2.0 billion people globally are infected with soil-transmitted helminths (STH) [1]. Soil-transmitted helminth infections, including with roundworm (*Ascaris lumbricoides*), whipworm (*Trichuris trichiura*), and hookworm species (*Necator americanus* and *Ancylostoma duodenale*), have been shown to be associated with growth retardation [2–5], impaired cognitive development [6, 7], and anemia and vitamin A deficiency [4, 8–11]. The burden of infection is borne disproportionately by preschool-aged children (PSAC) and school-aged children (SAC) [12].

Explosive urban growth has occurred in the last century, and for the first time in human history more people are residing in cities than in rural areas [13]. There has been a corresponding increase in urban slum growth, according to the United Nations Human Settlements Programme. As of 2007, approximately 1.0 billion individuals live in urban slums, and that number is expected to climb to 1.4 billion by 2020 [13]. Urban slums are characterized by overcrowding, poor-quality housing, and inadequate access to basic municipal services such as water and sanitation [14]. While evidence suggests that urban residents in general have better health outcomes and lower STH infection compared to their rural counterparts [15, 16], urban centers also likely represent heterogeneous areas of risk, and urban slum residents may be at higher risk than residents of adjoining non-slum urban areas for diseases affected by water, sanitation, and hygiene (WASH), such as STH infections [15, 17].

Preventative chemotherapy with anthelmintic drugs has long been considered the staple of STH control activities [18]. Increasingly, however, the importance of high-quality WASH in the control of STH is being recognized, as children in endemic settings cannot maintain a worm-free state with periodic deworming alone [19]. Access to and use of WASH have been shown to be associated with a reduced risk of transmission of STH infection in various settings [20, 21]; however WASH characteristics are complex and may be site-specific, particularly in urban settings such as Kibera.

Despite the increased emphasis on the role of WASH in STH control, gaps still exist in our understanding of the relationship between WASH and STH infection, particularly in slum areas. Therefore, further characterization of WASH factors and their impact in high-density urban settings is needed to identify the most appropriate and effective interventions.

The aim of this study was to simultaneously evaluate a comprehensive list of WASH risk factors for their association with STH infection among PSAC and SAC in an urban slum in Kenya. We used a cross-sectional STH prevalence survey conducted from April through June 2012 in Kibera, an urban slum in Nairobi, Kenya as an opportunity to collect an array of WASH and STH infection data among PSAC and SAC [22].

## Methods

### Ethics Statements

This study was approved by the ethical review committee of the Kenya Medical Research Institute (KEMRI) (KEMRI Protocol #2214) and by the Centers for Disease Control and Prevention (CDC) (CDC Protocol #6216) under a reliance agreement between the two institutions. Written informed consent was obtained from the parent or guardian of each enrolled child included, along with written assent from all participants aged ≥13 years. The results of stool examinations were provided to participants; treatment per WHO and Kenyan national guidelines was provided free of charge for all participants who were tested positive for any STH infection.

### Study Area

This study was conducted in Gatwekera and Soweto villages within the Kibera slum in Nairobi, Kenya. This area is characterized by semipermanent housing, high population density, and limited access to municipal water and sanitation services. Since 2005, the CDC’s International Emerging Infections Program (IEIP) and KEMRI have collaborated on community-based surveillance in this setting, consisting of bi-weekly household assessments for major infectious disease syndromes [23–25]. An up-to-date surveillance registry comprising approximately 28,000 participants is maintained by IEIP.

### Survey Design and Participants

Details on the methodology of participant selection are described elsewhere [22, 26]. Briefly, the sample was calculated to detect an odds ratio (OR) of 2.0 for STH infection associated with having an infected sibling living in the household, assuming 80% power and accounting for 20% non-response. The resulting sample size was 293 PSAC and 899 SAC, for a total of 1,192 children. To ensure that selected PSAC and SAC did not come from the same households, 25% of the households within the IEIP registry were designated as households for selection of PSAC participants and 75% of households were designated as households for selection of SAC participants. Then, households were selected from the list of designated households with probability proportional to number of PSAC or SAC population in the household. One PSAC (aged 6 to 59 months) or SAC (aged 5 to 14 years) was subsequently randomly chosen from each selected household.

### Data Collection

Field work for this study took place from April through June 2012. Trained study personnel, comprising community interviewers who conduct bi-weekly surveillance activities, approached selected households and invited them to participate. A household key informant, preferentially the female head of household was asked to participate in a comprehensive household-level structured questionnaire and an environmental assessment for WASH characteristics. The interview included questions about household demographics, composition, construction, expenditures, and possessions, as well as child-specific information including school attendance and past-year deworming treatment. Also, the child was asked to report if he or she had received deworming medication at school in the last year. To assess WASH status, study personnel conducted an extensive household assessment using both self-reported and observational metrics. This assessment used and expanded upon the WHO and United Nations Children’s Fund (UNICEF) Joint Monitoring Programme for Water Supply and Sanitation (JMP) core questions on drinking-water and sanitation for household surveys [27].

Water-related questions included the type and location of the primary household water source, water collection time, water availability and perceived ability to meet water needs, and household water treatment and storage practices. Sanitation questions included regularly-used sanitation facilities, household sharing of the sanitation facility, location and condition of the sanitation facility, and disposal of infant feces. Hygiene questions included availability of hygiene facilities near the sanitation facility, handwashing timing, soap ownership and use, location of handwashing facilities, availability of handwashing supplies, water dispensing method, and demonstration of handwashing technique. Environmental questions, assessed based on observations of the household environment and surroundings by study personnel, included: household construction materials, presence of electricity, presence of sewage or stool in the surrounding environment, and household location assessed by global positioning system (GPS).

We attempted to collect three morning stool specimens from each enrolled child. Stools were only accepted if produced after midnight on the day of collection. Subsequently, the collected stool samples were transported in cool boxes to the Eastern and Southern Africa Centre of International Parasite Control (ESACIPAC) laboratory at KEMRI by 14:00 the same day where the stool was immediately tested for STH ova (*A*. *lumbricoides*, *T*. *trichiura*, and hookworm) using the Kato-Katz method [28]. For each stool sample, two slides were prepared and read within 60 minutes by a trained microscopist to identify the STH species and to quantify the eggs. Positive sample results were converted into species-specific eggs per gram (EPG) of stool, and the mean EPG for each child was categorized as a light, moderate, or heavy intensity infection according to WHO definitions [29]. Children with ≥1 STH species identified in stool were also classified as having ‘any STH’ infection. Approximately 7% of slides were re-read by a senior microscopist for quality control purposes; final decisions of discordant reads were reached based on consensus.

### Statistical Analysis

Criteria for inclusion in the analyses included providing consent, and assent if applicable, not having withdrawn from the study, providing at least one analyzable stool sample, and having a completed household questionnaire and environmental assessment. The household’s primary water and sanitation sources were classified in accordance with the JMP water and sanitation ladders [30]. Dichotomous variables were created for many continuous variables; multi-category variables were collapsed into dichotomous variables due to small cell counts.

Full details on the sampling weighting procedure can be found elsewhere [22]. Briefly, sampling weights were calculated by inverting the initial selection probability and then multiplying by a weighting class adjustment and post-stratification weight to account for non-response and oversampling by IEIP sector, respectively.

Basic summary statistics incorporate sampling weights. Univariable and multivariable models were fit using the survey package [31] in R version 3.0.1 (R Core Team, 2014). The least absolute shrinkage and selection operator (LASSO) implemented in the glmnet package was used to select variables for a multivariable model [32]. Cross-validation was used to choose the shrinkage parameter. All variables listed in S1 Table were considered for selection in the multivariable model and no variables were forced in the final model. Final models incorporated the sampling weights, although results with unweighted models were similar. A separate, multivariable model of restricted to hygiene-related predictive variables was created using the same approach as above. Poisson regression was used for all models and results are reported as prevalence ratios. The 5% level of significance was used for all statistical tests.

### Factor Analysis for Socio-economic Status

Exploratory factor analysis was used to find latent variables describing socioeconomic status (SES) among variables concerning household possessions, cooking, and expenditures. A promax rotation was used to simplify the factor structure [33]. Variables with a loading of 0.7 or higher in absolute value were considered to load highly to a latent variable.

### Elevation Interpolation

**Fig 1:**
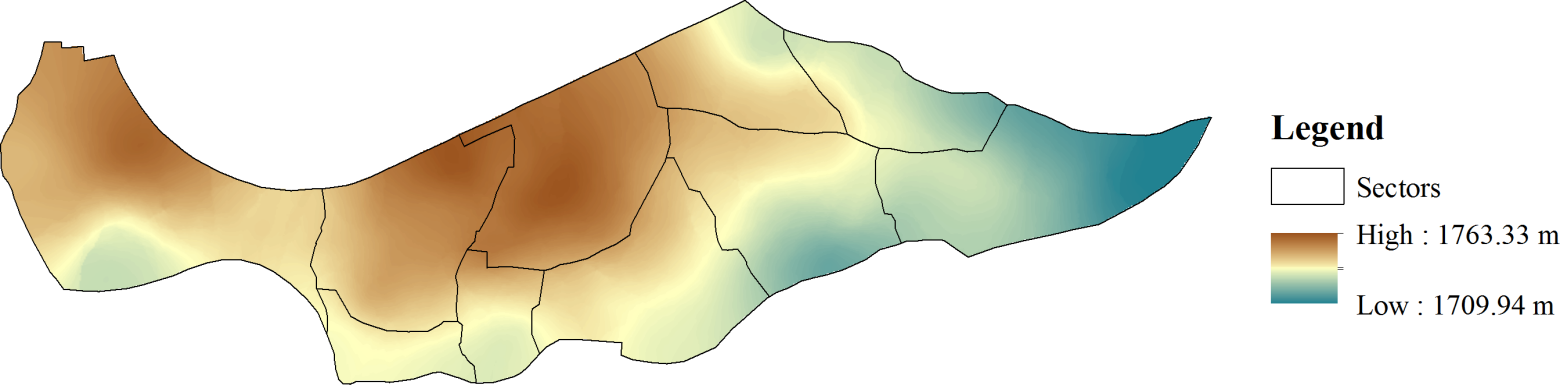
Household elevation surface interpolation generated using kriging method.

Household GPS points were collected at the time of the survey. Elevation readings were collected from October to November 2012 at some households due to missing or poor quality GPS data in the initial survey. GPS coordinates with a dilution of precision greater than 5.0 were considered to be of poor quality. Additional altitude data were collected at households requiring GPS readings for other IEIP purposes and from other landmarks in Kibera. A total of 449 readings were subsequently mapped using ArcGIS Version 10.1 (ESRI, Redlands, CA). A smooth elevation surface was created from this set of readings using kriging (Fig 1)[34]. Elevation points were extracted from the smooth surface for each household. The study site is situated along a shallow valley where household elevation ranged from 1,709 to 1,764 meters, and was categorized in 10 meter intervals.

## Results

**Fig 2:**
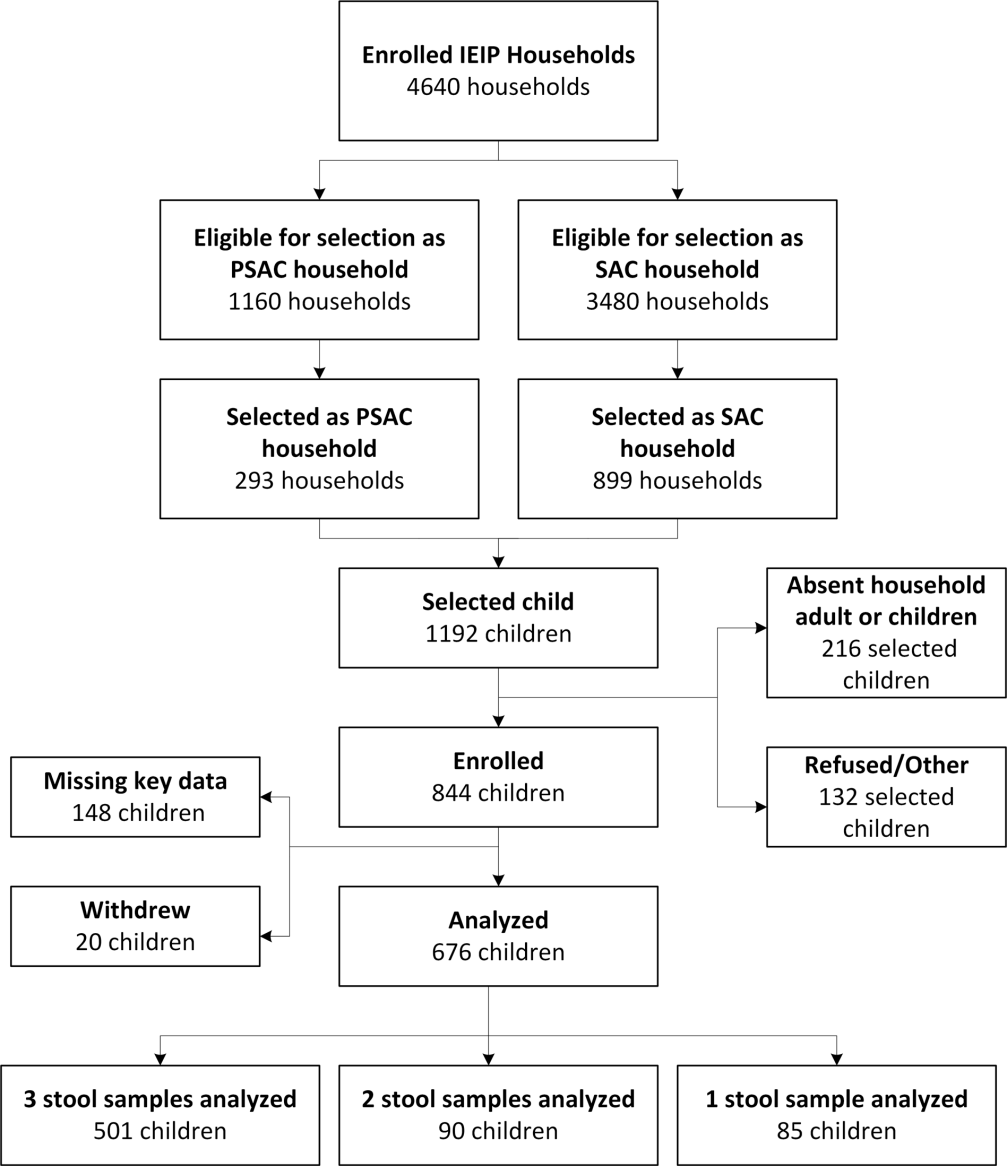
Participant flowchart showing participant selection, inclusion and analysis 2012.

Out of a targeted 1,192 children, a total of 844 children were enrolled in the study (Fig 2).

In order to be eligible for analysis, individuals were required to submit at least one analyzable stool and have a completed household survey. A total of 676 households containing either a selected PSAC (n = 201) or SAC (n = 475) were analyzed. Frequencies of examined demographic, child-specific, and WASH exposures as well as bivariate results of those exposures are presented in Table 1.

**Table 1:** Bivariate Associations for Selected Water, Sanitation, and Hygiene-related Risk Factors with Any Soil-Transmitted Helminth Infection among PSAC and SAC Children.

Characteristic	Observations	Frequency (%) / Mean (SD)	Unadjusted PR	95% CI
**Household demographics**				
Female head of household education level (years)	676	8.20 (2.4)	0.97	0.94–1.01
Ethnicity	676			
Kisii–Reference		40 (5.9%)	-	-
Luhya		123 (18.2%)	1.55	0.83–2.91
Luo		469 (69.4%)	1.95	1.08–3.51*
Other		44 (6.5%)	1.57	0.78–3.19
Household crowding (individuals/bedroom)	676	5.09 (2.0)	1.01	0.96–1.06
Wealth				
Factor 1 –Animals	676	N/A	0.96	0.87–1.06
Factor 2 –Cooking	676	N/A	0.95	0.85–1.05
Factor 3 –Household possessions	676	N/A	0.94	0.83–1.06
**Child characteristics**				
Age (years)	676	8.96 (4.43)	0.99	0.97–1.01
Female sex	676	360 (53.3%)	0.97	0.80–1.16
Dewormed in the last year	676	380 (56.2%)	0.80	0.66–0.96*
Always wears shoes	676	189 (28.0%)	0.80	0.64–1.01
Attends nursery or school	676	555 (82.1%)	1.30	1.05–1.62*
**Water indicators**				
Main water source	676			
Piped water into dwelling, plot, or yard–Reference		10 (1.5%)	-	-
Other improved		666 (98.5%)	3.32	0.54–20.39
Unimproved		0 (0%)	-	-
Water collection time > 60 min	669	12 (1.79%)	1.36	0.78–2.39
Weekly household water expenditures (USD)	676	$2.15 ($1.92)	0.91	0.69–1.21
Weekly household water expenditures per person (USD)	676	$0.38 ($0.32)	0.96	0.91–1.01
Constant water availability	665	96 (14.4%)	1.14	0.89–1.45
Difficulty meeting water needs	676	480 (71.0%)	1.20	0.96–1.49
Water stored in household	675	599 (88.7%)	0.90	0.68–1.18
Narrow mouthed storage container (3 cm or less)	595	381 (64.0%)	1.02	0.83–1.25
Storage container covered	593	518 (87.4%)	0.75	0.58–0.96*
Storage container on floor	592	445 (75.2%)	1.18	0.92–1.52
Ever treats water	676	411 (60.8%)	0.87	0.72–1.05
Always treats water	676	256 (37.9%)	0.81	0.66–0.99*
Treats water frequency	676	411 (60.8%)	0.87	0.72–1.05
Always–Reference		256 (37.9%)	-	-
Usually		84 (12.4%)	1.58	1.23–2.04**
Sometimes		67 (9.9%)	0.80	0.52–1.21
Never		269 (39.8%)	1.24	1.00–1.54
**Sanitation**				
Main sanitation facility	676			
Improved		9 (1.33%)	0.31	0.05–1.93
Shared–Reference		578 (85.0%)	-	-
Unimproved		68 (10.1%)	1.15	0.87–1.51
Open Defecation		21 (3.2%)	1.01	0.59–1.73
Improved or shared sanitation facility (Day)	675	590 (87.4%)	0.90	0.70–1.17
Improved or shared sanitation facility (Night)	673	428 (63.6%)	0.86	0.71–1.03
Shares toilet facility	673	642 (95.4%)	1.25	0.74–2.10
Toilet used by at least 10 other households	673	424 (63.0%)	0.90	0.74–1.09
Toilet located outside household premises	675	557 (82.5%)	1.33	1.00–1.77*
Feces observed in toilet facility	641	439 (68.5%)	1.09	0.88–1.35
Sewage observed pooling from latrine	642	93 (14.5%)	1.00	0.76–1.31
**Hygiene**				
Washed hands today or yesterday	675	657 (97.3%)	0.88	0.53–1.47
Used soap today or yesterday	675	606 (89.8%)	0.91	0.68–1.21
“When do you wash your hands?”	675			
After defecating/using the toilet		608 (90.1%)	1.01	0.74–1.37
Before preparing food or cooking		628 (93.0%)	1.23	0.81–1.89
Before feeding children		60 (8.9%)	0.83	0.58–1.20
Handwashing station characteristics				
Water dispensed into basin	369	353 (95.6%)	2.60	0.72–9.33
Water available	436	355 (96.4%)	1.39	0.60–3.24
Cleanser available	436	326 (87.6%)	0.92	0.62–1.36
Towel available	371	71 (19.1%)	0.69	0.45–1.04
Handwashing station available near toilet	644	117 (17.7%)	1.19	0.95–1.50
Handwashing demonstration				
Washed hands 30 seconds	486	223 (45.9%)	0.96	0.83–1.11
Rubbed hands together at least 3 times	486	466 (95.9%)	0.82	0.51–1.32
Washed with water	486	484 (99.6%)	1.39	0.60–3.24
Used soap	486	405 (83.3%)	0.81	0.63–1.06
Dried hands	486	298 (61.3%)	0.98	0.78–1.23
Dried hands on garment	486	157 (32.3%)	1.08	0.86–1.37
Dried hands in air	486	45 (9.3%)	1.22	0.88–1.70
Dried hands with towel	486	96 (19.8%)	0.75	0.55–1.04
Dried hands with clean towel	484	78 (16.1%)	0.58	0.38–0.87**
**Environment**				
Elevation (10 meter increments)	676	1,740 (12.0)	0.89	0.83–0.96**
Finished household flooring material†	676	566 (83.7%)	0.76	0.62–0.94**
Electricity in the home	676	602 (89.1%)	0.70	0.55–0.87**
Feces observed in yard	675	159 (23.6%)	1.14	0.93–1.41
Sewage observed in yard	676	49 (7.3%)	1.09	0.78–1.52

* p-value <0.05

** p-value ≤0.01

†—Finished flooring material includes vinyl/asphalt strips, cement, or carpet

### STH infection

Among the 676 children included in this analysis, 501 (74.1%) provided three stools, 90 (13.3%) provided two, and 85 (12.6%) provided one. Prevalence of infection with any STH (*A*. *lumbricoides*, *T*. *trichiura*, or hookworm infection) was 40.2% overall, with 40.8% and 40.0% of PSAC and SAC infected, respectively. Infection with *A*. *lumbricoides* (23.2%) and *T*. *trichiura* (26.5%) was most common, while hookworm infection was rare (0.2%). Concomitant infection with *A*. *lumbricoides* and *T*. *trichiura* was seen in 64 participants (9.5%). Heavy STH infection with any species was seen in 1.6% of participants.

### Household demographics

The majority of the surveyed population was of Luo ethnicity (69.4%). A higher prevalence of STH infection was seen in Luo households compared with other ethnic groups (prevalence ratio (PR) = 1.95; 95% Confidence Interval (CI) = 1.08–3.51). The average household size was 6.1 (standard deviation (SD) = 2.3) individuals, with an average of 5.1 (SD = 2.0) individuals residing per sleeping room, a metric for household crowding [35].

To assess household socio-economic status, we asked a series of questions regarding household characteristics extracted and adapted from the Kenyan Demographic and Health Survey [36]. Factor analysis revealed three latent factors which, based on the factor loadings, included animal ownership (Factor 1), cooking methods (Factor 2), and household possessions and expenditures (Factor 3).

### Child characteristics

The average age of the selected children was 9.0 years (SD = 4.4) and 53.3% of the children were female. Among children selected, 82.1% currently attend nursery, primary, or secondary school. In all, 56.2% of children were reported to have received anthelminthic medication in the last 12 months from a variety of sources, described elsewhere [22, 37]. Deworming in the last 12 months was significantly associated with a reduction in STH infection prevalence (PR = 0.80; 95%CI = 0.66–0.96).

### Water

In total, 1.5% of participants reported having piped water on premises (inside the home or plot) and 98.5% reported using another improved drinking water source, mainly public taps (97.6%). However 67.7% of water sources were deemed on observation to be clandestine self-connections to adjacent municipal pipes. The vast majority of households reported an inconsistent water supply and difficulty meetings household water needs, usually due to financial barriers. In Kibera, where water is often purchased at water kiosks, average household weekly water expenditure was $2.15 USD (SD = $1.92) with an average per capita water expenditure of $0.38 USD per week. Water was stored in the household in 88.7% of households. Further, 60.8% of respondents reported ever treating their household drinking water, while 37.9% reported always treating their drinking water.

Two water indicators were significantly associated with any STH infection in the bivariate analysis (Table 1). Children living in households where drinking water was always treated had a lower prevalence of STH infections than children living in households where drinking water was not always treated (PR = 0.81; 95%CI = 0.66–0.99). When children living in households with always-treated water were compared with those in households that usually treated, always treating still had a protective effect (PR = 1.58 in usually-treating households; 95%CI 1.28–2.04).

Further, children living in households where their water storage vessel had a cover had a reduced prevalence of any STH infection compared with children living in households where the vessel was not properly covered (PR = 0.75; 95%CI 0.58–0.96). However, neither the size of the vessel’s opening nor the storage location was significantly associated with STH infection.

### Sanitation

Of all households surveyed, 1.3% had access to improved sanitation facilities, 81.7% shared sanitation facilities, and 13.9% accessed unimproved sanitation facilities; 3.1% of households practiced open defecation (e.g., no sanitation facilities). Most households shared their sanitation facility with at least one other household (95.4%) with more than half of households sharing their facility with 10 or more other households. A higher proportion of respondents reported that their household members practice open defecation or only had access to unimproved facility at night (36.4%) than during the day (12.6%).

For most households (82.5%), the primary sanitation facility was located outside the household premises. Only 18.2% of households had a latrine connected to the house, whereas 70.3% used a facility located ≤50 meters from the house, and 11.5% used a facility located >50 meters from the house.

Conditions of toilet facility construction and cleanliness were assessed. Sewage was visibly pooling from the toilet on the surrounding ground in 14.5% of toilet observations. Human or animal feces were observed on the floor, slab, seat, or walls inside 68.5% of toilet facilities.

Children who lived in households where the primary sanitation facility was located outside the household premises had a higher prevalence of STH infection than those who lived in households where the sanitation facility was located on the household’s premises (PR = 1.33; 95% CI 1.00–1.77).

### Hygiene

Of all 675 adult respondents, 657 (97.3%) reported washing their hands either on the day of the interview or the day before the interview. Respondents reported washing their hands after defecation (90.1%), before preparing food or eating (93.0%), after eating (42.2%), and before feeding children (8.9%).

The surveyor made a discreet observational assessment of the households’ main handwashing station. Of 676 respondents, 436 (64.5%) were willing and able to show the surveyor the main handwashing station. Nearly all respondents demonstrated pouring water into a basin and washing hands in the water poured into this basin (95.6%) as their primary handwashing steps. Water and cleansers (e.g. soap or detergent) were available 96.4% and 87.6% of assessed handwashing stations, respectively. A towel was available at 19.8% of handwashing stations.

After the surveyor assessed the handwashing station, participants were asked to demonstrate handwashing techniques. Some individuals were able to demonstrate handwashing even though the surveyor was unable to assess their handwashing station, due to handwashing materials being stored out of plain sight. Only 486 (71.9%) participants were willing and able to demonstrate typical handwashing techniques to the surveyor. When asked to perform a handwashing demonstration, most participants wet their hands with water (99.6%), applied a cleansing agent (84.0%), and rubbed their hands together at least 3 times (95.9%). Hand drying after cleansing was less common (61.3%) with participants using a variety of drying tools including a garment (32.3%), the air (9.3%), and a towel (19.8%). Among those who dried their hands with a towel, the towel was assessed qualitatively for cleanliness; 81.2% of these individuals dried their hands using a towel that was considered clean by the surveyor.

Children living in households where mothers dried their hands using clean towels during the handwashing demonstrations had a lower prevalence of STH infection than children living in households where mothers dried their hands in any other manner or did not dry them (PR = 0.58; 95%CI 0.38–0.87).

### Environment

Several aspects of the domestic and peri-domestic environment were assessed as possible risk factors for STH infection. The construction material(s) of the household’s roof, walls, and floor were assessed. The household walls were frequently (94.2%) constructed of a mixture of rudimentary materials (i.e. stick with mud, uncovered adobe, or corrugated iron), though some (5.8%) were constructed with finished wall material. The roofing material was almost exclusively (99.9%) constructed of corrugated iron, known locally as *mabati*. Finally, 15.8% of households had a natural floor (i.e., earth or sand), 0.4% had a rudimentary floor (i.e., wood planks or palm/bamboo/sticks), and 83.7% had a finished flooring material (i.e., vinyl/asphalt strips, ceramic tiles, cement, and carpet). Electricity was available in 89.1% of homes.

Several environmental indicators were significantly associated with STH infection (Table 1). First, children living in a household where the main floor material was finished (i.e., vinyl/asphalt strips, ceramic tiles, cement, and carpet) had a lower prevalence of STH infection than children living in a household with natural flooring material (i.e., earth or sand) (PR = 0.76; 95%CI = 0.62–0.94). In addition, children living in households with electricity had a lower prevalence of STH infection than children living in households that lacked electricity (PR = 0.70; 95%CI = 0.55–0.87). Finally, children living in households at lower elevation had higher prevalence of STH infection; for every 10 meter increase in elevation, the prevalence of STH infection decreased by approximately 10% (PR = 0.89; 95%CI 0.83–0.96).

### Multivariate and Hygiene Sub-analysis

Multivariable Poisson regression was undertaken to explore the potential associations between WASH characteristics and STH infection. The complete multivariable model is presented in Table 2. Many associations identified in bivariate analysis remained important in multivariable analysis and no new variables emerged. Variables associated with STH infection included being dewormed in the last year (adjusted prevalence ratio (aPR) = 0.76; 95%CI = 0.63–0.92); 10 meter increments of elevation (aPR = 0.90; 95%CI = 0.83–0.97); usually *versus* always treating drinking water (aPR = 1.53; 95%CI = 1.15–2.01); and having a finished *versus* any other flooring material in the household (aPR = 0.76; 95%CI = 0.60–0.97).

**Table 2:** Associations for Water, Sanitation, and Hygiene-related Risk Factors with Any Soil-Transmitted Helminth Infection among PSAC and SAC Children in Multivariate Analysis.

Variable	Adjusted PR (95% CI)	P value
Age (years)	0.99 (0.97, 1.01)	0.37
Female sex	0.98 (0.81, 1.19)	0.87
Luhya ethnicity	1.40 (0.75, 2.99)	0.29
Luo ethnicity	1.67 (0.94, 2.99)	0.08
Other ethnicity	1.51 (0.76, 3.01)	0.24
Household crowding (individuals/sleeping room)	0.99 (0.94, 1.04)	0.68
Dewormed in last year	0.76 (0.63, 0.92)	<0.01*
Factor 2 –Cooking	0.97 (0.86, 1.09)	0.66
Elevation (10 meters)	0.89 (0.83, 0.97)	<0.01*
Water collection time (15 minute intervals)	1.01 (0.96, 1.06)	0.73
Water treatment frequency–Usually	1.52 (1.16, 2.01)	<0.01*
Water treatment frequency–Sometimes	0.68 (0.43, 1.06)	0.09
Water treatment frequency–Never	1.21 (0.97, 1.53)	0.09
Distance from house to sanitation (inside or connected) vs. (26–50 meters)	1.11 (0.79, 1.55)	0.55
Distance from house to sanitation (less than 10 meters) vs. (26–50 meters)	1.08 (0.79, 1.48)	0.63
Distance from house to sanitation (1–25 meters) vs. (26–50 meters)	1.15 (0.85, 1.54)	0.37
Distance from house to sanitation (> 50meters) vs. (26–50 meters)	1.04 (0.71, 1.54)	0.81
Availability of handwashing near toilet	1.22 (0.94, 1.58)	0.12
Water expenditures (USD)	0.99 (0.94, 1.05)	0.71
Presence of finished flooring material in home	0.76 (0.60, 0.97)	0.03*
Female head of household years of education	0.99 (0.95, 1.03)	0.70
Child always wears shoes	0.84 (0.66, 1.08)	0.18

* p-value <0.05

Due to the absence of data from handwashing demonstrations, several hygiene variables were systematically missing and it was not possible to include these variables in the overall model. Therefore, a multi-variable hygiene sub-analysis was fit separately. The multivariable model chosen by the LASSO procedure included two variables associated with STH infection: drying hands with a clean towel was associated with reduced STH prevalence (aPR = 0.60; 95%CI = 0.40–0.92), while presence of handwashing facilities near the toilet facility was associated with an increased prevalence (aPR = 1.20; 95%CI = 0.92–1.56).

## Discussion

Approximately 40% of both PSAC and SAC in this setting were infected with at least one species of STH. In endemic areas, STH infection has been shown to rebound to at least half of the initial infection prevalence by six months post-treatment [38], as treatment of children cannot eliminate eggs or worms in the environment and hence re-infection occurs easily. We investigated the association of WASH and environmental factors with STH infection status in children to identify possible opportunities for intervention.

### Water

The ability to access a sufficient quantity of high-quality water for meeting daily drinking, hygiene, and general household needs is critical. With limited officially-provisioned water services [39], hundreds of individual water vendors operate in Kibera, collecting water from networks of legal and illegal pipes connected to water mains that run through or near Kibera [40]. Most individuals purchase water, sometimes at great expense [39–41], from these vendors and transport water back to the household where it is stored until use. In our survey, nearly two-thirds of individuals surveyed reported having difficulty meeting daily household water needs. Inconsistent availability of water at the source, financial constraints on water purchase, and lack of water storage facilities are challenges reported in this setting that may impact the quantity and quality of water available to the household.

While ingestion of contaminated water is not considered a typical route of STH infection, previous research has demonstrated the presence of STH eggs in water samples [42, 43]. *Ascaris lumbricoides* eggs can remain alive in cold water for months [44]. The risk of contamination of water with pathogens is present both at the site of water draw and when water is being transported and stored within a household [45]. Previous work has explored the site of contamination by comparing pathogens present at the site of water draw with those found in water stored in household water storage containers [44, 46]. Khairy *et*. *al*. provide evidence that while helminth ova were absent from water coming from a tap, they were present in samples drawn from the water storage container, suggesting the possibility of contamination during the transport, storage, or usage of the water [44].

We examined the effect of household water treatment and safe storage on STH prevalence. Our results showed a decreased prevalence of STH infection among children living in households where water is always treated. Our findings were compatible with those of a meta-analysis by Strunz *et*. *al*. which showed that, across multiple studies, using treated water was associated with a lower likelihood of STH infection [21]. Our results mirror findings from other diseases where the effect of water treatment on preventing infection is dependent on compliance with treatment protocols [47, 48]; imperfect compliance diminishes the benefits derived from drinking water treatment.

We also evaluated several aspects of water storage systems including the storage container opening, cover, and storage location. Our results demonstrated that children living in households with covered water storage vessels have a lower prevalence of any-STH infection than those living in households with inadequate or absent water storage vessel covers. The protective effect of having a cover or lid on a water container has similarly been seen in diarrheal disease research in Bangladesh [49].

We were unable to perform a robust evaluation of the association between the JMP water source categories and STH infection because no individuals reported relying on an unimproved water source. Piped water access has been shown to be correlated with reduced prevalence of *A*. *lumbricoides* and *T*. *trichiura* infections [21]. Water source type may remain a factor associated with STH infection and should be explored further in the future.

### Sanitation

Access to improved sanitation available in Kibera is restricted by temporal, spatial, financial, and other obstacles. Our data highlight the temporal obstacles to improved sanitation as the number of household respondents reporting using unimproved sanitation facilities or practicing open defecation was significantly higher at night than during the day. In addition to the limited operating hours of many public toilet facilities, an additional barrier during evening hours (for women and girls) may be the risk of sexual assault and rape [50]. Further, 83% of respondents reported a spatial barrier between individuals and their sanitation facility: it may simply be too difficult to take the time to reach the facility at all times. Financial constraints may also encourage families to ration use of public sanitation facilities. One common alternative for households in the absence of using other sanitation facilities is the use of the ‘flying toilet’, where individuals defecate in a polyethylene bag and dispose of the bag in the environment [41]. If the bag ruptures and its contents are exposed to other people while STH ova are still viable, STH transmission could result.

Even during the hours when the public toilets are open, other barriers to access to improved sanitation exist. Less than two percent of households have access to an improved primary sanitation facility, defined by JMP as a private facility that ensures hygienic separation of human excreta from human contact [30], primarily because they share it with at least one other household. Most reported sharing their household toilet with ≥10 other households. With an average household size of approximately six persons, this corresponds to at least 60 users per toilet; similar figures are reported elsewhere [41, 50]. Tumwabaze *et*. *al*. found a negative association between the number of households sharing the sanitation facility and the satisfaction with the facility [51].

Access to and use of an improved toilet facility alone may not be sufficient to ensure the separation of human excreta from human contact. Many of the toilet facilities in Kibera are neither connected to the municipal sewerage system nor accessible for vehicles to empty fecal sludge. Therefore, gravitational emptying, whereby the contents of the pit latrine are emptied directly into a nearby river or drain where they flow down the elevation gradient, is a common practice, particularly during the rainy season when sludge can be washed away by rainwater [52]. Unsafe fecal sludge management strategies such as this undoubtedly introduce excreta into the immediate environment. Fecal sludge analysis from public toilets in similar settings have shown the concentration of 20,000–60,000 helminth eggs per liter of fecal sludge [53].

### Hygiene

Proper hand hygiene, including wetting, lathering, scrubbing, rinsing, and drying the hands [54], is one of the most critical components of disease prevention. Most participants in our evaluation reported recently having washed hands and were compliant with guidance regarding wetting and lathering with a cleanser. However, most participants washed and rinsed their hands in a basin of standing water rather than using the recommended running water [54–57].

Hand drying is a critical—though often underemphasized—component of hand hygiene. Our results show decreased prevalence of STH infection among children whose caregiver dried his/her hands using a clean towel compared with those who did not dry their hands or dried their hands on clothing or air drying. Further context on hand drying practices in Kibera can be found elsewhere [58]. Previous research has shown that the benefits of hand drying may be achieved through both the reduction in residual moisture that can act as a conduit for pathogen transfer and encourage bacterial growth and through physical removal of microorganisms through friction [56, 59–63], while sub-optimal hand drying practices may re-contaminate hands [60]. However, research examining optimal hand drying techniques, particularly in resource-limited settings, is limited and findings are inconsistent [60].

### Environment

Fecal contamination of the domestic and peri-domestic environment may increase risk of STH infection, as shown by Pullan *et*. *al*. [64]. In our study, increased prevalence of STH infection was seen among children living in households with unfinished (e.g., dirt) floors. While unfinished floors have been seen to be associated with hookworm infection [64–66], there is less evidence to support this association in *A*. *lumbricoides* and *T*. *trichiura* [67, 68]. Unfinished floors may be more difficult to clean, leading to the accumulation of pathogens, and may more easily aerosolize these pathogens, leading to the contamination of the household environment.

While absolute elevation has been shown to influence the viability of helminth eggs in the environment and therefore STH prevalence [69], our analyses suggest that relative elevation may also be important for STH infection. Open drainage channels are often used as a disposal site for solid waste (e.g. flying toilets) and fecal sludge as described above. This may lead to accumulation of pathogens at lower elevations in the slum consequently putting those residents at increased risk of STH infection. This association has been seen similarly in typhoid fever in children in Kibera [70].

Several limitations of this study are noted. Due to space constraints in this setting, many households do not have dedicated handwashing stations; instead, many households use a multi-purpose basin for handwashing activities in addition to other household activities. This led to many missing observations as the materials used for handwashing may not have been evident to the surveyor or they were not able to observe the handwashing station. Also, reported WASH variables may be subject to social desirability bias and the handwashing demonstration may be subject to the Hawthorne effect [71].

Further, there was frequently a homogenous distribution of explored exposures, limiting the ability to examine these factors. For instance, wealth and socio-economic status is often associated with STH outcomes [72–74], but none of our wealth outcomes were associated with STH in univariable analyses or selected for the multivariable model. We hypothesize that the small variability in wealth across the study area contributes to the lack of association between wealth and STH infection. Further, it may not be possible to extrapolate these findings to the remainder of the Kibera slum or slums more generally. Households that participate in IEIP surveillance have access to free medical services at a local clinic, and therefore health behaviors and outcomes might not be typical. However, this could suggest that the prevalence and associations reported here are conservative estimates compared with other slum locations. Finally, it is also important to note that the figures presented in this analysis, particularly for prevalence, differ slightly from other reports arising from this study due to slight differences in requirements for inclusion in analysis [22, 26].

The intersection between water, sanitation, and hygiene sectors and soil-transmitted helminth infection is complex. Insecure tenure, absentee landlords, and poverty hinder infrastructure improvements in areas such as Kibera. Despite these challenges, NTD programs should consider at least basic WASH interventions to sustain the gains made by deworming activities. These WASH interventions should be tailored to site-specific conditions to have the biggest impact.

## Supporting Information

S1 Table Complete list of Bivariate Associations for Water, Sanitation, and Hygiene-related Risk Factors with Any Soil-Transmitted Helminth Infection among PSAC and SAC Children. (PDF)
