# Supplementary material for: A Cross-Sectional Study of Water, Sanitation, and Hygiene-Related Risk Factors for Soil-Transmitted Helminth Infection in Urban School- and Preschool-Aged Children in Kibera, Nairobi
Source: PLoS One. 2016 Mar 7;11(3):e0150744. doi: 10.1371/journal.pone.0150744 (PMC4780697; doi:10.1371/journal.pone.0150744)
Supplement: S1 Table — (PDF) [file pone.0150744.s001.pdf]

Supplemental Table 1. Complete list of Bivariate Associations for Water, Sanitation, and Hygiene-related Risk Factors with Any Soil-Transmitted Helminth Infection among PSAC and SAC Children

Characteristic	Observations	Frequency (%)/ Mean (SD)	Unadjusted PR	95% CI	P value
**Household demographics**					
Female head of household education level (years)	676	8.20 (2.4)	0.97	0.94-1.01	0.09
Ethnicity	676				
Kisii – Reference		40 (5.9%)	-	-	-
Luhya		123 (18.2%)	1.55	0.83-2.91	0.17
Luo		469 (69.4%)	1.95	1.08-3.51	0.03*
Other		44 (6.5%)	1.57	0.78-3.19	0.21
Household Crowding (individuals/bedroom)	676	5.09 (2.0)	1.01	0.96-1.06	0.69
Wealth					
Factor 1 – Animals	676	N/A	0.96	0.87-1.06	0.44
Factor 2 – Cooking	676	N/A	0.95	0.85-1.05	0.30
Factor 3 – Household possessions	676	N/A	0.94	0.83-1.06	0.32
**Child characteristics**					
Age (years)	676	8.96 (4.43)	0.99	0.97-1.01	0.46
Female sex	676	360 (53.3%)	0.97	0.80-1.16	0.72
Dewormed in the last year	676	380 (56.2%)	0.80	0.66-0.96	0.02*
Always wears shoes	676	189 (28.0%)	0.80	0.64-1.01	0.06
Attends nursery or school	676	555 (82.1%)	1.30	1.05-1.62	0.02*
**Water indicators**					
Main water source	676				
Piped water into dwelling, plot, or yard - Reference		10 (1.5%)	-	-	-
Other improved		666 (98.5%)	3.32	0.54-20.39	0.20
Unimproved		0 (0%)	-	-	-
Water collection time > 60 min	669	12 (1.79%)	1.36	0.78-2.39	0.28
Weekly household water expenditures (USD)	676	$2.15 ($1.92)	0.91	0.69-1.21	0.52
Weekly household water expenditures per person (USD)	676	$0.38 ($0.32)	0.96	0.91-1.01	0.16
Constant water availability	665	96 (14.4%)	1.14	0.89-1.45	0.31
Difficulty meeting water needs	676	480 (71.0%)	1.20	0.96-1.49	0.10
Water stored in household	675	599 (88.7%)	0.90	0.68-1.18	0.43
Narrow mouthed storage container (3 cm or less)	595	381 (64.0%)	1.02	0.83-1.25	0.86
Storage container covered	593	518 (87.4%)	0.75	0.58-0.96	0.02*
Storage container on floor	592	445 (75.2%)	1.18	0.92-1.52	0.19
Ever treats water	676	411 (60.8%)	0.87	0.72-1.05	0.14
Always treats water	676	256 (37.9%)	0.81	0.66-0.99	0.04*
Treats water frequency	676	411 (60.8%)	0.87	0.72-1.05	0.14
Always - Reference		256 (37.9%)	-	-	-
Usually		84 (12.4%)	1.58	1.23-2.04	<0.001 *
Sometimes		67 (9.9%)	0.80	0.52-1.21	0.29
Never		269 (39.8%)	1.24	1.00-1.54	0.05
**Sanitation**					
Main sanitation facility	676				
Improved		9 (1.33%)	0.31	0.05-1.93	0.21
Shared - Reference		578 (85.0%)	-	-	-
Unimproved		68 (10.1%)	1.15	0.87-1.51	0.32
Open Defecation		21 (3.2%)	1.01	0.59-1.73	0.97
Improved or shared sanitation facility (Day)	675	590 (87.4%)	0.90	0.70-1.17	0.43
Improved or shared sanitation facility (Night)	673	428 (63.6%)	0.86	0.71-1.03	0.10
Shares toilet facility	673	642 (95.4%)	1.25	0.74-2.10	0.40
Toilet used by at least 5 other households	673	468 (69.5%)	0.93	0.77-1.12	0.43
Toilet used by at least 10 other households	673	424 (63.0%)	0.90	0.74-1.09	0.27
Improved infant stool disposal	658	307 (46.7%)	0.95	0.78-1.14	0.57
Toilet located outside household premises	675	557 (82.5%)	1.33	1.00-1.77	0.047*
Feces observed in toilet facility	641	439 (68.5%)	1.09	0.88-1.35	0.42
Sewage observed pooling from latrine	642	93 (14.5%)	1.00	0.76-1.31	0.99
Toilet component in good condition					
Roof	642	527 (82.0%)	0.83	0.66-1.04	0.11
Slab[¥]	590	543 (92.0%)	0.88	0.62-1.23	0.45

Door	642	563 (89.2%)	0.89	0.68-1.16	0.38
**Hygiene**					
Washed hands today or yesterday	675	657 (97.3%)	0.88	0.53-1.47	0.63
Owns soap	676	620 (91.7%)	0.84	0.63-1.13	0.26
Used soap today or yesterday	675	606 (89.8%)	0.91	0.68-1.21	0.50
"When do you wash your hands?"	675				
After defecating/using the toilet		608 (90.1%)	1.01	0.74-1.37	0.97
Before preparing food or cooking		628 (93.0%)	1.23	0.81-1.89	0.33
Before feeding children		60 (8.9%)	0.83	0.58-1.20	0.33
Handwashing station characteristics					
Water dispensed into basin	369	353 (95.6%)	2.60	0.72-9.33	0.14
Water available	436	355 (96.4%)	1.39	0.60-3.24	0.44
Cleanser available	436	326 (87.6%)	0.92	0.62-1.36	0.59
Towel available	371	71 (19.1%)	0.69	0.45-1.04	0.08
Handwashing station available near toilet	644	117 (17.7%)	1.19	0.95-1.50	0.14
Handwashing demonstration					
Washed hands 30 seconds	486	223 (45.9%)	0.96	0.83-1.11	0.57
Washed both hands	486	478 (98.4%)	0.66	0.37-1.19	0.17
Rubbed hands together at least 3 times	486	466 (95.9%)	0.82	0.51-1.32	0.42
Washed with water	486	484 (99.6%)	1.39	0.60-3.24	0.44
Used cleanser	486	408 (84.0%)	0.84	0.64-1.10	0.19
Used soap	486	405 (83.3%)	0.81	0.63-1.06	0.12
Dried hands	486	298 (61.3%)	0.98	0.78-1.23	0.87
Dried hands on garment	486	157 (32.3%)	1.08	0.86-1.37	0.49
Dried hands in air	486	45 (9.3%)	1.22	0.88-1.70	0.24
Dried hands with towel	486	96 (19.8%)	0.75	0.55-1.04	0.09
Dried hands with clean towel	484	78 (16.1%)	0.58	0.38-0.87	<0.01*
**Environment**					
Elevation (10 meter increments)	676	1,740 (12.0)	0.89	0.83-0.96	<0.01*
Finished household flooring material†	676	566 (83.7%)	0.76	0.62-0.94	0.01*
Electricity in the home	676	602 (89.1%)	0.70	0.55-0.87	<0.01*
Household floods during rain	676	248 (36.7%)	1.00	0.82-1.21	0.98
Feces observed in yard	675	159 (23.6%)	1.14	0.93-1.41	0.20
Sewage observed in yard	676	49 (7.3%)	1.09	0.78-1.52	0.63
Sewage observed in neighbour's yard	676	122 (18.1%)	0.94	0.73-1.20	0.63

* p-value <0.05

** p-value ≤0.01

† - Finished flooring material includes vinyl/asphalt strips, cement, or carpet

¥ - The quality of slab was not assessed on facilities that were not designed to include a slab (i.e., pit latrine without a slab)
